# A review for cell-based screening methods in drug discovery

**DOI:** 10.52601/bpr.2021.210042

**Published:** 2021-12-31

**Authors:** Fen Wei, Sicen Wang, Xilan Gou

**Affiliations:** 1 Health Science Center, School of Pharmacy, Xi’an Jiaotong University, Xi’an 710061, China

**Keywords:** Cell-based screening, Drug candidate, Microfluidics, Biosensor, Affinity chromatography

## Abstract

With the biological relevance of the whole cells, low cost compared with animal experiments, a wide variety of cell-based screening platforms (cell-based assay, cell-based microfluidics, cell-based biosensor, cell-based chromatography) have been developed to address the challenges of drug discovery. In this review, we conclude the current advances in cell-based screening and summary the pros and cons of the platforms for different applications. Challenges and improvement strategies associated with cell-based methods are also discussed.

## INTRODUCTION

**Figure 1:**
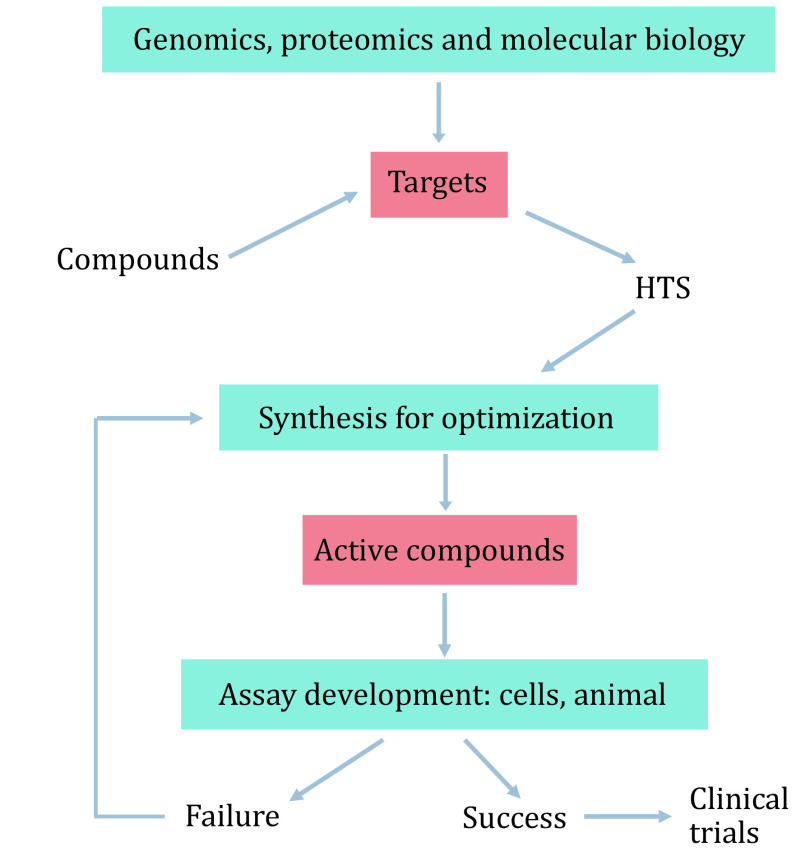
Lead generation with HTS screening

Traditional drug discovery involves a serial stage for the development of the new drug. It is expensive and can take 10–15 years. Mostly, high-throughput screening (HTS) is carried out after target confirmation, following with optimization of the compound structure, animal testing, and finally clinical trials (Fig. 1). However, it remains a high failure rate in drug discovery, which causes the tendency to discover new targets for drug repurposing for more diseases (Moridani and Harirforoosh 2014; Parvathaneni* et al.* 2019; Wang 2018). And the critical issue is the appropriate target (druggability of the target) that should provide an unambiguous, therapeutically significant response to improve the drug discovery (Jorgensen 2012; Roy 2019).

Actually, a more important reason is the lack of biological context during the screening process. In Arduino’s study, cryopreserved mitochondria isolated from yeast strain were engineered with functional protein and then were employed as a ready-to-use screening reagent. The reduced false discovery rate was carried out by energizing mitochondria with D-lactate in a mannitol/sucrose-based medium, which indicates the significance of bionic and *in vivo* environment (Arduino* et al.* 2021). Besides, among the anti-cancer drugs, sorafenib and regorafenib show significant differences in activity, but only one difference in structure for the non-hydrogen atom (*i*.*e*., a fluorine). So there is no sufficient resolution in current methods to distinguish compounds with subtle structural changes except for animal or cell assays (Schlessinger* et al.* 2017). It means new tools and techniques that can better reflect the *in vivo* environment are required during the drug discovery process.

Currently, the demand of cell-based functional assays in HTS is increasing. One obvious advantage is that cell-based assays present more physiologically relevant systems for the screening of compounds (Fursov *et al*. 2005). It indicates that cell-based screening has more potential for development (Szabo* et al.* 2017). For example, at the beginning of the genomic era, enzyme-based biochemical screens were focused during the antibacterial drug development to replace the traditional cell-based phenotypic screens. However, after a long period of HTS practice, it was discovered that the required drugs could not be successfully provided. Thereafter, the focus in the antibiotic drug discovery field has shifted back to whole cell-based phenotypic screens directly (Yuan* et al.* 2021). Kumar found that the result of screening against PanC which is considered a druggable target had no significant cellular activity in a variety of biochemical screens. In contrast, traditional whole-cell screening has proven more successful. The reason may be multiple new targets can be implemented on the whole cell (Kumar* et al.* 2017). The discovery of antibiotics is mainly through cell-based screens, as the inhibit activity of identified novel inhibitors based on essential enzymes targets was not as expected (Datta 2021). Screening in whole cells can reveal a great deal more about the targets and action mechanism of compounds compared to* in vitro* screening based on enzyme or protein targets (Adamson* et al.* 2021).

Given the importance of biological context, preclinical models are widely used in drug discovery, including* in vitro* models (cell culture), *ex vivo* models, and *in vivo* models (artificial, transgenic, non-transgenic and induced) (Shi* et al.* 2019; Xu* et al.* 2021). However, more than 20,000 molecules were screened using different animal models for Alzheimer’s disease drug development during the past two decades, only Aducanumab was approved by FDA (Cacabelos* et al.* 2021). The challenge is that no single model faithfully reproduces all the features of human disease. So, drug discovery can integrate different important attributes in a multisystem model, which can be achieved by cell-based models (Cacabelos* et al.* 2021; Kumar* et al.* 2017; Szabo* et al.* 2017).

Cell-based screening in drug discovery is usually two-dimensional (2D) screening, due to that 2D cell culture models remain the accepted standard for drug screening *in vitro*. 2D cell culture model can provide valuable insights into biological processes and effects of new drugs with low cost and efficient workflows, which is widely used in various screening methods (Amelian* et al.* 2017; Thippabhotla* et al.* 2019). However, growing evidence indicates that 2D cell culture models often fail to represent the underlying biology of cells, such as *in vivo* extracellular matrix microenvironment, and therefore cannot accurately predict the *in vivo* drug response (Belfiore* et al.* 2021; Godugu and Singh 2016). This review will summarize the current state relating to different cell-based screening technologies containing 2D and 3D models. It will also provide recent perspectives about the cell-based HTS from natural herbs in drug discovery.

## CELL-BASED ASSAY

The cell-based assay is usually combined with HTS, and the distinction between a cell-based assay and an *in vitro* screening is that the cell-based assay utilizes live cells seeded onto the floor of the well (Rajalingham 2016). Usually, cellular screening relies on different strategies ranging from reporter gene technology to protein fragment complementation assays. In order to reduce the response time, the monitoring of its first activation step can be treated as alternative approach by using fluorescence and bioluminescence resonance energy transfer (Michelini* et al.* 2010). Cell-based assays are used to identify the best drug candidate (Capula* et al.* 2019), measure proliferation (Adan* et al.* 2016), toxicity (Li* et al.* 2006), motility (Sanookpan* et al.* 2021), analyze cell signaling pathways (Pathe-Neuschafer-Rube* et al.* 2021), and changes in morphology (Rajalingham 2016). Among the cell-based assays, 2D versus 3D culture might also contribute to the results obtained.

### 2D screening of cell-based assay

A promising tool to bridge between species or from health to disease is *in vitro* cell culture. The simplest 2D models include monolayer cell culture, adding molecules or molecular libraries to the culture medium, and measuring the output with a microplate reader or microscope (Foster* et al.* 2021). Although they lack the sophisticated tissue structures or biophysical stimulation present *in vivo*, the way in which monolayer culture responds to chemical or genetic stress is largely consistent with clinical observations or primary cell data (Tu* et al.* 2021). In addition, a key advantage of a 2D model is the compatibility with high-throughput analysis. So, a simple 2D *in vitro* model may serve as a preliminary screening tool. Of course, the drawbacks of animal experiments such as extremely time-consuming and cost-intensive, a significant discrepancy between animal toxicity and human toxicity, are an aspect that promotes the development of cell-based assay (Doke and Dhawale 2015; Madden* et al.* 2020).

Conventionally, 2D models are performed in dishes, tubes, or well plates. The aim is to confirm the effect of the different concentrations of the candidate on cellular growth and function (Hamon* et al.* 2013; Hu* et al.* 2015). For the most widespread cell viability or cytotoxicity assays in drug discovery, 96, 384, or even 1,536 microtiter plates are most commonly used with colorimetric readouts of cell supernatants (Riss 2005; Wegener 2015). Radnai *et al*. presented a simple cell-based method for the discovery of novel cytokinesis inhibitors. The assay was performed in a 96-well plate format in 48 h. Then, living cells, nuclei and nuclei of dead cells are identified by a single staining step using three fluorescent dyes, followed by rapid live cell imaging (Radnai* et al.* 2020). Scaling up of screening systems, with the use of multiwell plates and multichannel pipettes (or even robotic liquid handling systems) is fairly commonplace. It should be noted that when using a multiwell plate, the number of cells per well and equilibration period before the assay will affect the responsiveness to compounds (Riss 2005). Heinzman *et al*. developed a liquid handler equipped with a 1000-μL capacity 96-tip tool for cell plating automate to minimize human error while increasing accuracy, precision, and efficiency (Heinzman *et al*. 2010). Soman *et al*. used plates that seeded with disialoganglioside (GD2) — expressing cell lines to bind and screen the anti-GD2 molecules and quantify the GD2-specific binding activities. They found that the cell-based assay showed more consistent and reproducible comparing with microtiter plate coated with purified GD2 (Soman* et al.* 2011). Thomas *et al*. developed a rotatable disc microfabricated with multichannel for performing cell growth and cell-based assays in a liquid medium. The apparatus and methods can be used to measure a variety of biochemical processes and products. Combining with non-invasive techniques does not compromise the integrity or viability of cells (Thomas 2011).

In terms of detection on cell-based assays, improvements in various detection techniques are also promoting the development of cell-based methods. A new plate reader (Nanotaurus) was developed by Edinburgh Instruments, which has the principal features of a confocal microscope and acquires data by the technique of time correlated single photon counting. This instrument demonstrates the advantages of biochemical assays and shows strong promise for cell-based assays (Näther *et* *al*. 2006). The microscopic imaging technique is the necessary detection method for many cell-based assays, but due to the cost of equipment, it is not in general widely adopted for primary screening. So Olson *et al*. used enzyme complementation to provide an analytical method that uses substrates to generate luminescent signals. The principal advantage of this method is amenable to HTS using microtiter plate protocols (Olson and Eglen 2007). Mohiuddin *et al*. stably co-expressed target fragments tagged with luminescence probes in HEK-293FT cells and identify five compounds as lead compounds (Mohiuddin* et al.* 2021). Fluorescent imaging often requires the removal of background fluorescent signals to obtain robust measurements, which is challenging for high-density microplates. In view of this problem, a wash-free cell-based fluorescence assay method was proposed, which uses a laser scanning fluorescence plate cytometer. This work shows that sensitivity and efficiency are increasing while assay artifacts are reduced, and results in the development of broadly applicable cell-based fluorescence imaging assays for drug screening (Gorshkov* et al.* 2020).

Mainly primary animal cells, tissue specimens, and immortalized as well as tumor cell lines have been used in cell-based assays (Fritsche* et al.* 2021). Most cell-based screening is often engineered to overexpress targets or reporter constructs, due to that the immortalized cell lines are easy to culture and expand, which is quite suitable for HTS. For example, *Spodoptera frugiperda* insect cell expressed hCOX-1 and hCOX-2 proteins was used to identify the selective inhibitors of hCOX-1 and/or hCOX-2 (Zhang* et al.* 2004). However, the generation of cell lines involves the cell clones by proliferating *ex vivo* which is different from the *in vivo* counterparts. Its experimental condition may alter growth characteristics and signal transduction pathways. By contrast, primary cells are more closely reflect cell behaviors in human tissues and more physiologically relevant to human biology (Berg 2019; Berg* et al.* 2014). Tumor cell lines are another type of primary cells, and more closely reflect the genetic and clonal heterogeneity of the native tumor *in vitro* model system, thus providing a more accurate pre-clinical platform (Corallo* et al.* 2020). Wang *et al*. found human lactate dehydrogenase A (hLDHA) is overexpressed in osteosarcoma cells as compared to a human normal cell. So they used a cell-based phenotypic screening assay to solve the highly polar nature of hLDHA, and discovered three cellular active inhibitors (Wang* et al.* 2020a).

Simple 2D cell-based assays have limitations, partly due to their plate format. So a wall-less plate technology was present, which takes advantage of hydrophobic and hydrophilic surface properties of the unique liquid. This technology showed an obvious advantage when suspension cells were used in multistep experimental procedures (Quinones* et al.* 2013). Some groups sought to introduce an extra level of complexity to increase the physiological relevance of their 2D screening systems. Another mean was to introduce an extracellular matrix to mimic chemical and mechanical properties, which was designed for the screening models of tissue types (Foster* et al.* 2021). Zhang *et al*. first described the differentiation of hESCs into a mixed culture of neurons, astrocytes, and oligodendrocytes (Zhang* et al.* 2001). From 2D cell culture-based monolayers, multilayer to co-culture models, their aims were to promote physiological characters, reproducibility and mimic characteristic functionalities of disease modeling (Kutlehria and Sachdeva 2021). In order to develop *in vitro* models, many factors need to be considered, such as cell line type, cell culture medium, substrate roughness and stiffness. They affect the final outcome of the* in vitro* assay through the significantly effect of the microenvironment. Advanced technologies based on 3D models have allowed the development of more complex structures, bridging the gap between *in vitro* and *in vivo* models (Yuste* et al.* 2021).

### Limitations of 2D format

Although simple models are easier to create and faster to reproduce, their systems present a number of limitations. Some candidate molecules often fail to perform *in vivo*. One reason is that the 2D models lack microenvironments, such as complex geometrical architecture, paracrine signals from neighboring cells, mechanical properties, nutrition and oxygen, to mimic the native tissue. This microenvironment will strongly influence cellular behavior and functionalities containing proliferation, differentiation and metabolism (Berg 2019; Davoudi* et al.* 2021; Rimann and Graf-Hausner 2012; Wollrab* et al.* 2016). On the other hand, enhanced drug sensitivities are proved in 2D conditions and require lower dosage ranges, resulting in ineffective *in vivo* (Foster* et al.* 2021). In cell-based assays, a main hurdle is to design a sufficiently powerful detection method with adequate signal to noise while maintaining the inherent physiology of the cells (Halim 2020).

### 3D screening model

Improving the success rate in the early stages of drug development requires disease models with high biological relevance for biomarker discovery and drug development. In cell-based experiments, the rapid increase in 3D cell culture technologies more closely mimics *in vivo* physiology, which is considered a promising step to improve the success rate of drug discovery (Langhans 2021). Especially for tumor models, 3D format is similar to *in vivo* tumors, which can recapitulate the complexity of the tumor microenvironment, and therefore bridge the gap between 2D monolayers and animal models (Fontana* et al.* 2021). The 3D cell culture models either rely on the self-organizing properties of mammalian cells or use bioengineered constructs to arrange cells like the organ. A self-assembling 3D multicellular brain model is used to mimic the complex *in vivo* cytoarchitecture of the brain. The data showed that the combination of 3D cell culture and bioengineering can improve reproducibility and tissue architecture (Hattori 2014; Lancaster* et al.* 2017). Additionally, some studies create simple 3D co-culture models by using a mixture of cell types present in the tissue microenvironment to observe the responses *in vivo* (Belfiore* et al.* 2021; Lazzari* et al.* 2018).

The 3D cell models include spheroids, hanging drops, scaffolds, cell sheets, hydrogels, bioreactors, and microfluidic chips (Bialkowska* et al.* 2020; Yuste* et al.* 2021). The scaffold-free 3D cell models including multicellular tumor spheroid models are better in terms of *in vivo* context simulation compared to 2D cell models, but they are lack of extracellular matrix recapitulation that limits their applicability in relevant drug testing (Cavo* et al.* 2016). Scaffolds are widely used to create 3D models, such as collagen scaffold, chitosan-alginate scaffold, nanofiber scaffold and hydrogel scaffold (Leung* et al.* 2010; Liu* et al.* 2018b; Yang and Zhao 2011). The advanced technologies such as microfluidics, biosensor and chromatography will be described later.

### Successes from cell-based assay

**Figure 2:**
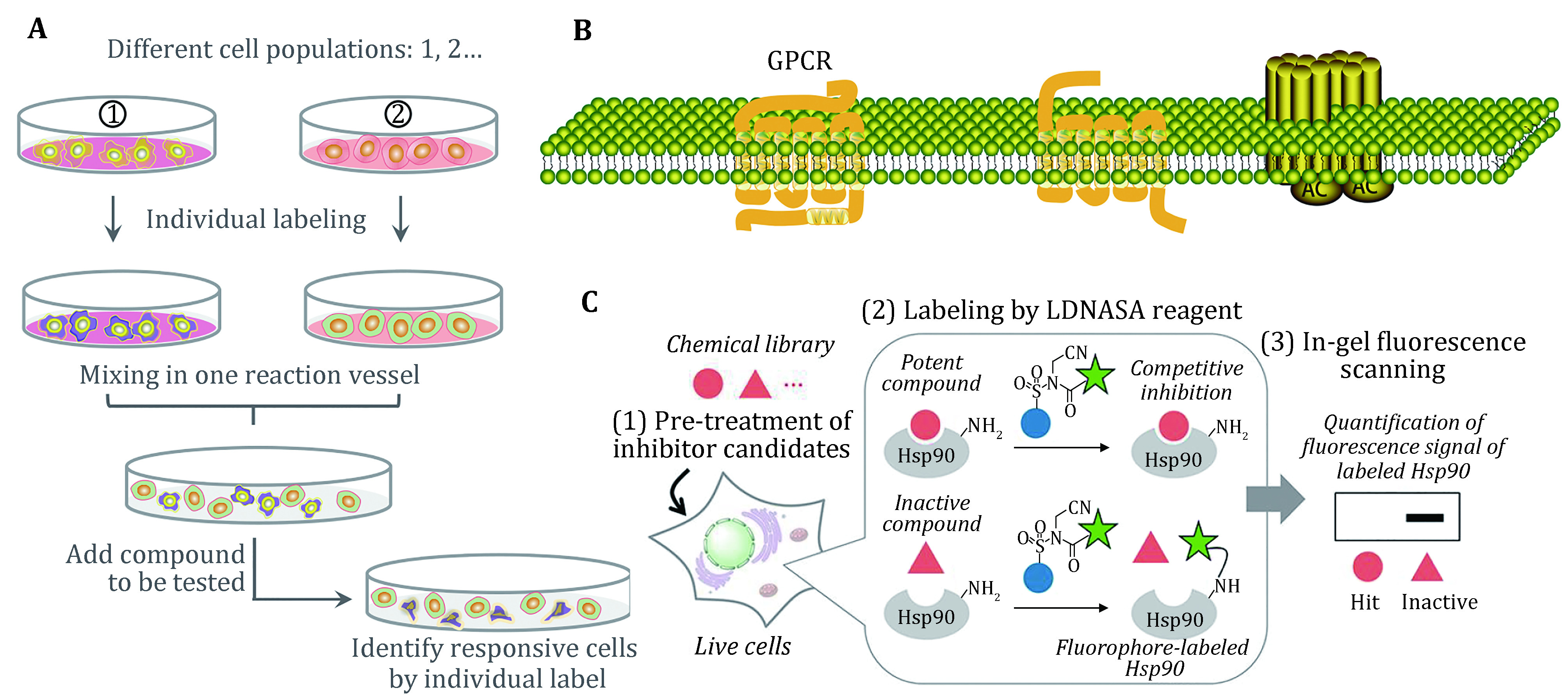
Scope of application for cell-based assay. **A** Unknown targets can use the whole cells for screening. **B** GPCRs as target that is difficult for purification. **C** Cell-based ligand-screening system for inhibitor or allosteric inhibitor (Ueda* et al.* 2020)

Cell-based assays are suit to screen targets that are refractory to biochemical purification and can characterize compounds with unknown targets (Fig. 2). In physiologically relevant settings, intracellular signals can be transmitted so that agonists and antagonists can be identified. Meanwhile, different binding sites of the same receptor, especially allosteric sites, can be screened for diverse pharmacological effects of compounds (An and Tolliday 2010; Drewe and Cai 2010; Berg* et al.* 2014; Zaman* et al.* 2007).

In the present study, some of the same compounds screened by different cell-based assays show different pharmacological activities. For example, brefeldin A can inhibit the cytotoxic effects of ricin (Wahome* et al.* 2010) and can also inhibit the growth of two pairs of parental and Pgp-overexpressing multidrug-resistant cell lines (Zahra* et al.* 2020). Apigenin stimulates hair growth through downregulation of the TGF-β1 gene (Huh* et al.* 2009) and is also identified as potent activators of PXR-mediated CYP3A4 promoter activation (Dong* et al.* 2010), activators of the JAK/STAT pathway (Tai* et al.* 2012). Quercetin can inhibit ABCG2 activity (Henrich* et al.* 2006) and prevent *H. pylori* adhesion and infection (Sekiguchi* et al.* 2008), also can be a potential IFN mimic or adjuvant in new antiviral drugs (Tai* et al.* 2012). Luteolin can prevent *H. pylori* adhesion and infection (Sekiguchi* et al.* 2008) and is also identified as ANO1 inhibitors as potential anticancer therapeutic agents for prostate cancer (Seo* et al.* 2017), besides, it is also identified as a potential IFN mimic or adjuvant in new antiviral drugs (Tai* et al.* 2012). In addition, Table 1 shows the active compounds screened by cell-based assay in the past five years that may be developed into promising drug candidates.

**Table 1:** Cell-based screening assay for candidate drugs

Cell type(s)	Model	Active compounds	References
MCF-7 Cells/ OR6M1-expressing cell lines	SPR chip immobilized cells. A modified carboxymethyl dextran sensor chip	Anthraquinone, rutin	Choi* et al.* 2021
DNA-PKcs and OCT4 - expressing HEK-293FT cells/ MK2 and OCT4 expressing NCI-H82 cells	384-well plate. Two-step method. Exogenously expressing proteinsin cancer cells as first step	A cardioglycoside and an isocarbostyril alkaloid, cholesterol-based structures (three compounds)	Mohiuddin* et al.* 2021
Vero E6 cells	96-well plates. Transfected 2-E plasmids after compounds pre-incubated	34 hits with cell protection activity	Wang* et al.* 2021
HeLa cells	24-well plates. Treated with compounds followed by labeling of the intracellular Hsp90. Analysis with in-gel fluorescence after cell lysis	157 compounds. Morin	Ueda* et al.* 2020
COS7 cells	Clean glass. Single-protein tracking in a living cell. Effects are evaluated by diffusion coefficient shift using fluorescence microscopy	Hyperoside for EGFR and ErbB2. DiAB-141 and 2”-O-acetylvitexin for ErbB3	Kim *et al*. 2021
COS-7 cells	96-well plates. Cells seeded in monolayer and molecules added to the medium	Blebbistatin, *para*-aminoblebbistatin, *para*-nitroblebbistatin, jasplakinolide, cytochalasin D, swinholide A	Radnai* et al.* 2020
HEK293:FLP-InT-REx-BiFC#20 cells	384-well clear-bottom, black-walled microplate. Cells seeded in monolayer and molecules added to the medium	6 compounds. Isocotoin	Xu* et al.* 2020
Vero E6/A549/Huh7/ LN-18 cells	6-well plate format and 96-well plate. Cells infected viral dilutions. Cells seeded in monolayer and antivirals in infection medium added to cells	Sofosbuvir and ribavirin	Vicenti* et al.* 2020
HEp-2/ A549/293T cells	96-well plate. Cells seeded in monolayer and molecules and virus added to the medium	Laby A1/A2	Blockus* et al.* 2020
Human embryonic stem cells/ Fibroblasts from healthy donors and patients	384-well plates. Cells seeded in monolayer and molecules added to the medium	CUDC-907	Kase* et al.* 2021
HpeG2/ HeLa cells	Culture chamber. 3D electric cell/matrigel-substrate impedance sensing chip. Cells seeded in prechilled matrigel solution and generated 3D structure	Taxol, cisplatin, sorafenib	Pan* et al.* 2019
HeLa cells	96-well plates and 384-well plates. Cells seeded in monolayer and molecules added to the medium followed with added viral solution	11 compounds. Gemcitabine	Zhang* et al.* 2017
MEFs	96-well plates. Cells seeded in monolayer and molecules added to the medium	Cantharidin, Nifedipine	Semenova* et al.* 2017
HeLa cells	384-well clear-bottomed black plates. Cells seeded in monolayer and molecules added to the medium	18 compounds	Hajjar* et al.* 2017
MDA-MB-231 breast cancer cells/ HUVECs	96-well plates. Cells seeded in monolayer and extracts added to the medium	Cirsimaritin, *Cirsium japonicum* extract, cirsimaritin	Yeon Park* et al.* 2017
HepG2 ARE reporter cells	384 well pate format. Cells seeded in monolayer and molecules added to the medium	AZ-628, PHA-767491, SL-327, PAC-1, pifithrin-α, vitamin B12	Liu* et al.* 2018a
MC3T3-E1-OSE cells	96-well plates. Cells seeded in monolayer and molecules added to the medium	4 compounds. T63	Zhao* et al.* 2017
ARPE-19 cells	96-well plate. Cells seeded in monolayer and molecules added to the medium	47 compounds	Maruyama* et al.* 2018
MG-63 cells	96-well plate. Cells seeded in monolayer and molecules added to the medium	42 compounds	Wang* et al.* 2020a
A549 cells	384-well plates. Cells seeded in monolayer and infected with DENV1-4, and treated immediately with compounds	(S)-29	Kounde* et al.* 2017
SPR: surface plasmon resonance; MEFs: NF1-deficient mouse embryonic fibroblasts			

## ADVANCED CELL-BASED SCREENING TECHNOLOGIES

### Microfluidics technologies for drug screening

Microfluidics is also known as Lab-on-a-chip, represents a technology that can precisely control and manipulate sub-millimetre scale fluids in geometry. In the last decades, microfluidic devices have gradually been used as a multi-functional tool for many types of cell-based analysis, such as in drug screening and discovery, cell culture, cell separation, intracellular signaling, toxicity and so on (Gupta* et al.* 2016). Microfluidic devices offer some benefits including rapid analysis, high sensitivity and reproducibility. Its key advantage is microscale dimensions that match with the cellular structures and microenvironments like the human body. Because of its nanoliter volumes samples and reagents, microfluidic technology is very cost effective. As with cell-based assay, microfluidic technology also can simulate the* in vivo* response. Especially, the miniaturization of microfluidics is suitable for HTS, compared with some cell-based assays (Caruso* et al.* 2020; Hattori* et al.* 2013).

In the application of high-throughput screening, three major complementary modes can be used to manipulate microfluidic. Perfusion flow mode requires a series of components to introduce reagents and samples, transferring and mixing fluids in the microchannel network. This mode manipulates the liquid flows continuously by external mechanical pumps or the capillary forces combined with electro-kinetic form (Coliaie* et al.* 2021; Hao *et al*. 2020). The liquid flows also can be driven by vacuum-driven pressure or gas-generating chemical reactions (Park* et al.* 2020). Gao *et al*. carried out one-step cell seeding and anti-cancer drug testing by a microfluidic channel combined with vacuum actuated chambers (Gao* et al.* 2013). Guler *et al*. developed a self-powered microfluidic device. The key part is a 3D-printed effervescent pump for CO_2_ generation from a chemical reaction. When the coagulation starts, an acid-base reaction is triggered for the gas generation that drives the fluids within the channels (Guler *et al*. 2018). Using gravity driven flow is another possible solution. Zhu *et al*. presented a gravity driven pumping system using arrays of horizontally-oriented mini-reservoirs to generate a constant flow rate across microﬂuidic channels (Zhu* et al.* 2004). The advantage of continuous-flow is easy implementation, which makes it to be the most widely accepted microfluidic platform for simple biomedical applications. However, there are some limits in the perfusion flow mode. The use of microchannels for continuous fluid delivery tends to result in higher reagent consumption. Moreover, when applied to large-scale drug screening, chip structures are often complex, involving multiple channels, liquid-controlled pump and valve designs (Liang* et al.* 2021).

Droplet mode always uses water-in-oil emulsion droplets to compartmentalize reagents into nanoliter to picoliter volumes. It will create unavoidable interface fluctuation during emulsification. It can encapsulate biomolecules into discrete droplets and uses the generated units for analysis. The droplets are usually generated by pressure-driven flow (Shembekar* et al.* 2016), containing hydrodynamics and pneumatic pressure. Electrowetting can generate droplets by surface tension drive (Lian 2019; Liu* et al.* 2021). Gravity-driven overflow microfluidic system can infuse fluids steadily and continuously, which requires less manual power (Gao *et al*. 2019). The hanging-drop platform used in the tissue model enables continuous inter tissue communication, constant medium turnover, and immediate exchange of metabolites by gravity-driven flow through the network (Boos* et al.* 2019). Droplets encapsulation can exclude sample loss on the surface wall by preventing the contact between the sample and the droplet wall. Comparing with continuous microfluidics, droplet-based microfluidic overcomes complex fluidic control, does not require separated channels for each sample, and minimizes dilution and contamination issues (Damiati* et al.* 2018). Its key characteristics are using a few microliters of samples and requiring few cells. Furthermore, a high degree of automation and ease of integration with HTS makes it very promising in drug discovery (Shembekar* et al.* 2016; Wang* et al.* 2020b). When droplet-based microfluidics is used to generate microcarriers, they exhibit the advantages of high drug loading and relatively long drug release. However, the formation of monodispersed carriers is not constant or repeatable due to the solvent evaporation and droplet solidification step. In particular, the formation of nano-sized carriers is limited by droplet-based microfluidic systems. Moreover, mechanical stirring will destroy the shape, morphology, size uniformity and loading efficiency of the droplets (Damiati* et al.* 2018).

Cell microarrays mode has been well established for cellular phenotypes investigation and offers invaluable advantages of HTS. This screening mode needs the generation of cell microarrays on a 2D solid substrate, and then applies drug combinations or drug libraries to those arrays (Li* et al.* 2018). Arrays can be composed of single cells, cell monolayers, aggregates or spheroids. Microarrays can screen for thousands of different samples simultaneously in one single experiment with low reagent consumption and high-content readouts. Although effective, their high cost and the requirement of specialized equipment for their manufacture limit their scope of application. Besides, cells cultured on the microarray can cause neighboring effects and cross-contamination (Du* et al.* 2016; Jonczyk* et al.* 2016; Zhang* et al.* 2016).

Microfluidic technology is an effective tool for the enhancement of drug discovery. But single cell analysis is mostly used for cell function research. The heterogeneous responses from individual cells can provide information at both the individual and population levels (Seah* et al.* 2018; Yin and Marshall 2012). As mentioned before, the 2D monolayer cell lacks the microenvironment, leading to the ineffective for disease. So, the combination of microfluidic technology with the 3D cell culture offers great potential for drug discovery (Liu* et al.* 2019). A microfluidic platform was developed for anticancer compound screening by using multicellular spheroids as a 3D model derived from tumor biopsies. The characters of this lab-on-a-chip platform are self-generating nutrients, drug concentration gradients perfusion and equipment-free (Mulholland* et al.* 2018). The supporting matrix or carrier for the 3D cell culture is an important factor in microdevices. It can be summed up as gel-supported 3D cell culture, gel-free 3D cell culture and gel-coated 3D cell culture. Gel-supported 3D cell culture allows the encapsulation of cells into the hydrogel, and permits oxygen permeability and nutrient transport. In order to mimic *in vivo* microenvironment, native extracellular matrix proteins are always used as the basis of hydrogel scaffolding, such as collagen, fibrin, fibronectin, hyaluronic acid, matrigel, agarose, poly(ethylene glycol) diacrylate, or a mixture of both. While for gel-free 3D cell culture, intercellular polymeric linker polyethylenimine-hydrazide, microwells, suspension or spheroids model can be selected to supplement the gel-supported 3D cell culture (Li *et al*. 2012).

### Cell-based sensor for drug screening

Cell-based biosensor systems consist of three components. The sensing unit contains cells for target identification. A transducer is used for converting biological reactions to chemical/electrical/optical signals, and the output system can amplify and readout signals (Zhou* et al.* 2011). It plays an outstanding role in drug discovery, cancer research and immunology. Cell-based biosensor systems that use whole cells as a living model have an obvious advantage, which is responding in a manner that can offer insight into the physiological effect of an analyte. The advantages include the detection of unknown compounds and toxins, readily coupling with HTS for drug candidates screening, and reducing the need for animal testing (Ozsoylu* et al.* 2021). In cell-based sensor detection, the key factors of cell function affected by the analytes can be singled-out without being disturbed by more complex, whole organism or whole organ responses. Cells grown in a thin layer have advantages in cell-based sensors, that is, they can be observed under a microscope or other optical equipment. Different cell types of cell-based sensors also show different advantages. For example, microorganism cells can be cultured easily and grow rapidly. It is less expensive to culture compared with mammalian cells. However, the mammalian cells can provide bioavailability and physiologic responses relevant to humans (Banerjee* et al.* 2010).

Since the cell-based biosensor uses living cells, its limitations are stability and robustness. On the one hand, researchers are trying to develop label-free biosensor technologies, which monitor the behavior of cells without stains damage or photobleaching effects (Shamah and Cunningham 2011). Due to the non-invasive nature of this technology, living cells can be continuously investigated, so real-time kinetic measurement can be achieved (Ona and Shibata 2010; Xi* et al.* 2008). Cryopreservation is another solution to maintain certain vital parameters of cells inside the sensor system. Özsoylu *et al*. proposed an on-sensor cryopreservation strategy with the modified chip surface. It can be effective for keeping cells viable on a biosensor chip (Ozsoylu* et al.* 2021). Due to the demand for high-throughput cellular assays, miniaturization of cell-based biosensors needs to be achieved by preparing cell microarrays. Flat substrates (positioning arrays) or particles (solution or suspension arrays) are used to immobilize different cells using various microfabrication technologies to achieve multiplexing and high-throughput cell-based sensing (Hong* et al.* 2017).

Despite the advantages of cell-based biosensors, some limitations are associated with the existing systems. Most cells used in the sensor are cultured on hard 2D glass or plastic matrix, which cannot mimic *in vivo* counterparts. Its weak cell-substrate attachment greatly shortens the effectiveness and life of cell-based biosensors (Mao and Kisaalita 2004). Advances in novel biomaterials and nano/micro engineering technologies have enabled to immobilize cells using scaffold-free 3D methods. So it is promising to address the limitation of 2D cell-based biosensors (Zhou* et al.* 2011). Dipeptide-derived hydrogel matrix was employed to encapsulate cells and enzymes that are used as sensing elements. This method is based on the self-assembly function of a small molecular hydrogel. An established 3D culture model based cellular biosensing system is useful for cellular function and drug discovery (Lian* et al.* 2017).

### Cell-based chromatography for drug screening

The technologies mentioned are not suitable for the HTS of complex systems like natural herbs. Natural products can be used to treat various diseases. For many years, plant-derived products have been recognized as sources of therapeutic agents and structural diversity (Chopra and Dhingra 2021). Nevertheless, natural products also present challenges for drug discovery, now we will introduce several improved analytical tools to open up the new opportunity (Atanasov* et al.* 2021). The chromatographic methods established by adsorbing cell membrane on the surface of silica gel to screen bioactive compounds from traditional medicines are lack of stability. So a new strategy was designed for attaching cells onto amino microspheres. The microspheres were prepared by coating poly (oligo (ethylene glycol) methacrylate) with RGD peptide using atom transfer radical polymerization. Then the cells were immobilized to the microspheres based on the specific affinity between integrin on the cells and the RGD peptide. This method can increase the density of cells in the stationary phase at the same time. As a result, three bioactive compounds were screened from *Ligusticum chuanxiong* using the established cell column (Li* et al.* 2015). Liu *et al*. developed a novel hollow fiber cell fishing procedure with high-performance liquid chromatography. These methods were used for rapid screening, fishing, and analysis of bioactive compounds from traditional Chinese medicines. Firstly, the cells were seeded on the internal surface of the fibers, followed by inserting into the extracts of herbs. The active compounds can be screened by cells inside the fibers. Finally, the active compounds were dissociated and analyzed using HPLC/MS (Liu* et al.* 2014). Although the screening process approximates the interaction between the bioactive component and the cells *in vivo*, the stationary phase cannot be reused due to the sensitivity of live cells inoculated on the fiber. Recently, we reported an innovative cell-based microcarrier chromatography to simulate *in vivo* drug-receptor interaction. Cells firstly grow on the microcarriers, then the attachment can be improved using paraformaldehyde. The success of paraformaldehyde fixation is based on a layer of denatured collagen on the surface of the microcarrier. Due to the use of microcarriers for 3D cell culture, the stationary phase loaded into the column also presents 3D characteristics. Combing with HPLC/MS, active compounds can be bionically screened and identified successfully (Wei* et al.* 2021). Although cell-based chromatography can more likely screen active lead drugs, it lacks the function of predicting cellular effects after screening and identification, and needs to combine with the cell-based assay for further activity verification.

## CONCLUSIONS

The need to increase clinically available drugs while reducing development costs is continuing to drive the development of cell-based screening methods. Each platform described in this review for drug discovery has associated strengths and limitations. In general, cell-based screening methods can build a bridge between animal experiments and human diseases. They are suitable to screen targets that are refractory to biochemical purification and characterize compounds with unknown targets. The screening results can be more physiologically relevant. Compared with animal experiments, cell-based screening methods are more efficient and less expensive. In addition, among these screening platforms, 3D models have more potential for drug development compared to 2D cell-based screening methods. Although numerous approaches exist today, it is very likely that a new strategy can combine several advantages of each approach in the future.

## Conflict of interest

Fen Wei, Sicen Wang and Xilan Gou declare that they have no conflict of interest.
